# Factor of time in dendritic cell (DC) maturation: short-term activation of DCs significantly improves type 1 cytokine production and T cell responses

**DOI:** 10.1186/s12967-024-05368-4

**Published:** 2024-06-06

**Authors:** Primož Poženel, Kaja Zajc, Urban Švajger

**Affiliations:** 1 Slovenian Institute for Transfusion Medicine, Šlajmerjeva 6, Ljubljana, 1000 Slovenia; 2 Faculty of Medicine, University of Ljubljana, Korytkova ulica 2, Ljubljana, 1000 Slovenia; 3 Faculty of Pharmacy, University of Ljubljana, Aškerčeva 7, Ljubljana, 1000 Slovenia

**Keywords:** Dendritic cells, Maturation, Vaccines, Cytotoxic T cells, Cancer, Anti-tumor responses

## Abstract

Dendritic cells (DCs) have been intensively studied in correlation to tumor immunology and for the development DC-based cancer vaccines. Here, we present the significance of the temporal aspect of DC maturation for the most essential subsequent timepoint, namely at interaction with responding T cells or after CD40-Ligand restimulation. Mostly, DC maturation is still being achieved by activation processes which lasts 24 h to 48 h. We hypothesized this amount of time is excessive from a biological standpoint and could be the underlying cause for functional exhaustion. Indeed, shorter maturation periods resulted in extensive capacity of monocyte-derived DCs to produce inflammatory cytokines after re-stimulation with CD40-Ligand. This effect was most evident for the primary type 1 polarizing cytokine, IL-12p70. This capacity reached peak at 6 h and dropped sharply with longer exposure to initial maturation stimuli (up to 48 h). The 6 h maturation protocol reflected superiority in subsequent functionality tests. Namely, DCs displayed twice the allostimulatory capacity of 24 h- and 48 h-matured DCs. Similarly, type 1 T cell response measured by IFN-γ production was 3-fold higher when CD4^+^ T cells had been stimulated with shortly matured DC and over 8-fold greater in case of CD8^+^ T cells, compared to longer matured DCs. The extent of melanoma-specific CD8^+^ cytotoxic T cell induction was also greater in case of 6 h DC maturation. The major limitation of the study is that it lacks in vivo evidence, which we aim to examine in the future. Our findings show an unexpectedly significant impact of temporal exposure to activation signals for subsequent DC functionality, which we believe can be readily integrated into existing knowledge on in vitro/ex vivo DC manipulation for various uses. We also believe this has important implications for DC vaccine design for future clinical trials.

**Supplementary Information:**

The online version contains supplementary material available at 10.1186/s12967-024-05368-4.

## Introduction

Dendritic cells (DCs) act at the central stage of our immunological network with the most important antigen-presenting cell (APC) function of the immune cell repertoire [1]. They are very efficient at processing antigens (Ags) and responding to environmental signals on one hand, while on the other, they can successfully transfer this gathered information to cells of the adaptive immune system. This results in orchestration of particular type (various kinds of immunogenic or tolerogenic) of Ag-specific responses [2]. By virtue of their »professional« APC characteristics, DCs have been used as biological tools for preparation of anti-cancer cellular vaccines in the clinic almost for the last three decades [3].

Initial clinical attempts were based on first-generation DC vaccines, where patient-isolated natural DCs or monocyte-derived DCs (MoDCs) were used without further manipulation of their activation state (maturation) [4]. Despite some success in early attempts, in general, the clinical responses using unmodified, immature DCs were very low. Along with increased understanding of immunological mechanisms behind generation of potent cytotoxic T lymphocyte (CTL) responses, required for efficient anti-cancer immunity, the importance of the maturation step in preparing DC-based vaccines was clearly evident [5]. With advances in the field this gave rise to second-, as well as next-generation DC vaccines, with documented clinical improvements [6].

Maturation of DCs is one of the most critical features of their biology and life-cycle [7]. Mechanistically, it represents a complex process designated by acquisition of several fundamental properties and is most frequently triggered by engagement of specific pattern-recognition receptors, namely toll-like receptors (TLRs) expressed by DCs. On a molecular level, DC activation via TLRs can trigger maturation via three main signaling pathways: mitogen-activated protein kinase (MAPK), nuclear factor-κB (NF- κB) and interferon regulatory factors (IRFs) [8]. This results in increased expression of several inflammatory genes and subsequently leads to significant improvement in their ability to induce both CD4^+^ T helper (Th) cell and CTL responses and has been shown as a prerequisite for inducing immune responses in cancer patients [9–11]. Moreover, DC maturation is a heterogeneous, finely-tuned process, which, depending on activation signals, their combination and time of exposure, can lead to different DC activation states with distinctive functional characteristics [12]. In terms of anti-cancer immunity, much effort has been made in the past toward optimizing type 1 DC maturation protocols, which enable high expression of co-stimulatory signals and particularly high production of Th1- and CTL-polarizing cytokines, especially interleukin (IL)-12 [13–15].

Although in general retrospect, the objective response rates (ORRs) to DC vaccines did not meet expectations and rarely exceeded 15%, the introduction of immune checkpoint inhibitors (ICIs) in the last decade with the possibility of combinatorial therapeutic approaches have revived the potential for incorporation of DC vaccines in cancer immunotherapy [6, 16]. This means that DC vaccines still constitute a promising treatment, with over 200 registered and active clinical studies currently registered on clinicaltrials.gov (accession date 20th February, 2024). Indeed, improvements in DC maturation potential with introduction of optimized protocols should represent a meaningful approach toward further advancements in clinical outcomes and combinatorial therapeutic approaches (e.g. ICIs). For this reason, further optimization of their efficacy is warranted, as any enhancement of their key functions for inducing anti-cancer immunity could hypothetically lead to subsequent improvements in their clinical translation.

With this in mind, we focused our current study on more or less neglected aspect of in vitro DC manipulation, namely the importance of temporal exposure of MoDCs to activation signals, for which there is currently a crucial gap in general knowledge. We demonstrate that for MoDCs to achieve their maximum stimulatory potential, the required duration for their exposure to maturation stimuli is much less than what is usually applied during in vitro or ex vivo protocols (24 h and more). Furthermore, we found that brief maturation (4–6 h) confers superior functionality in comparison to standard, 24–48 h (h) protocols, in their general capacity to induce type 1 immune responses.

## Materials & methods

### Isolation and culture of cells

Buffy coats were obtained from the venous blood of healthy volunteers following the guidelines of the Blood Transfusion Centre of Slovenia. The study was approved by the National Medical Ethics Committee (approval number 0120–279/2017-3). We isolated peripheral blood mononuclear cells (PBMCs) with gradient centrifugation using Lympholyte®-H from Cedarlane Laboratories in Ontario, Canada. Afterwards, we washed the cells twice with Dulbecco’s phosphate-buffered saline (DPBS). The cells were then counted and used for immunomagnetic isolation of CD14-positive monocytes using CD14 microbeads (Miltenyi Biotec GmbH in Bergisch Gladbach, Germany). Monocyte purity was consistently above 90%, as confirmed by flow cytometry.

To initiate differentiation of DCs, monocytes were cultured in Cellgenix® DC GMP medium from Cellgenix GmbH in Freiburg, Germany. The medium was serum-free and supplemented solely with 50 µg/ml gentamicin. Additionally, cell cultures were supplemented with 800 U/ml of rhGM-CSF and 1000 U/ml of rhIL-4 from Peprotech in London, UK. On day 2 and day 4, half of the culture medium was replaced, along with the initial amounts of rhGM-CSF and IL-4. On the 6th day, the immature DCs were collected, washed two times with DPBS, and counted on the Vi-Cell™ XR cell viability analyzer from Beckman Coulter (Fullerton, CA). For subsequent analysis or experiments, the immature DCs were either used as they were or exposed to a maturation cocktail. This cocktail consisted of 1 µg/ml of monophosphoryl lipid A (MPLA) from Invivogen and 1000 U/ml of IFN-γ. In certain instances, the DCs were also matured using a TLR3 agonist polyinosinic: polycytidylic acid (poly I: C, at 20 µg/ml) or a TLR8 agonist resiquimod (R848, at 2.5 µg/ml), both from Invivogen. The duration of exposure to the maturation stimuli varied depending on the specific experiment, ranging from 2 to 48 h as needed.

Human PBMCs were used to purify T cells. CD4^+^ T cells (bulk) were obtained through positive selection by utilizing CD4 microbeads (Miltenyi Biotec, GmbH). The purity of CD4^+^ cells consistently exceeded 90%, as determined by flow cytometry. We isolated naïve CD4^+^CD45RA^+^ T cells using the naïve CD4^+^ T-cell isolation kit (Miltenyi Biotec), adhering strictly to the manufacturer’s protocol. The purity of the isolated naïve CD4 + T cells consistently exceeded 95%. CD8^+^ T cells were isolated by employing CD8 microbeads (Miltenyi Biotec). The purity of the isolated CD8^+^ T cells consistently exceeded 90%.

### Apoptosis analysis

Viability of DCs was determined by analyzing the presence of early and late apoptotic cells by Annexin V-FITC and 7-aminoactinomycin (7-AAD) staining. Dendritic cells were either left untreated (immature DCs) or were matured with MPLA and IFN-γ for the longest observed period in our experiments (48 h). Measurements of viability were performed on a FACSCalibur flow cytometer (Beckton Dickinson).

### Phenotypic analysis

For the analysis of surface DC phenotype using flow cytometry, the following monoclonal antibodies (mAb) were used: FITC-labeled anti-CD14 and anti-HLA I (both from Invitrogen, Camarillo, CA), Alexa Fluor 488-labeled anti-CCR7 (Biolegend, CA, USA), PE-labeled anti-CD40, anti-CD80, anti-CD83, anti-CD86, and anti-HLA DR (Miltenyi Biotec, Bergisch Gladbach, Germany).

The DCs, which were differentiated in Cellgenix® DC GMP medium (Cellgenix GmbH, Freiburg, Germany), were harvested and collected through centrifugation. Prior to cell staining, the cells were washed two times in DPBS in all instances. The appropriate antibody was added, and the cells were incubated in the dark for 15 min. Subsequently, the cells were washed twice and suspended in 2% paraformaldehyde (PFA). Analysis of the samples was performed using a FACSCalibur system (Becton Dickinson, Inc.), and the acquired data were analyzed using the CellQuest software (BD biosciences).

### Cytokine detection after re-stimulation

Dendritic cells underwent differentiation and subsequent maturation using MPLA/IFN-γ for specific time intervals, as described previously. After 2 h, 4 h, 6 h, 8 h, 24 h, or 48 h, the DCs were collected and rinsed with DPBS. In some instances, the DCs were matured for 6 h and 24 h using poly I: C, R848 or a combination of both. Following the washing procedure, the cells were further stimulated for an additional 24 h through the CD40-CD40L pathway using the CD40-Ligand Multimer Kit (from Miltenyi Biotec, GmbH). This step aimed to mimic the interaction between DCs and T cells, as previously demonstrated [14]. Upon completion of the CD40L stimulation, the culture supernatants were gathered and assessed for the presence of TNF-α, IL-8, IL-6, IL-1β and IL-12p70, using the cytokine bead array methodology, following the manufacturer’s protocol (CBA assay; BD Biosciences, CA, USA). The samples were analyzed utilizing a FACSCalibur flow cytometer (BD biosciences, CA, USA). To determine cytokine concentrations in the supernatants, a standard curve was prepared through serial dilutions of standards.

### T cell proliferation assays

In co-culture experiments involving allogeneic T cells, dendritic cells (DCs) that had undergone differentiation in Cellgenix® DC GMP medium were employed as stimulators. The DCs were either maintained in their immature state or activated using MPLA (1 µg/ml) and IFN-γ (1000 U/ml) for durations of 6 h, 24 h, or 48 h. As responders, purified whole CD4^+^ T cells or purified whole CD8^+^ T cells were utilized. The assays were conducted in 96-well plates, with a total volume of 200 µl per well. For the co-cultures, we used RPMI 1640 growth medium (Lonza, Verviers, Belgium) supplemented with 10% human AB serum. A cell count of 2 × 10^4^, 1 × 10^4^ or 5 × 10^3^ was used for DCs, while 2 × 10^5^ cells were used for responder T cells, to achieve DC : T cell ratios of 1:10, 1:20 and 1:40, respectively. After 4 days, the wells were pulsed with 1 µCi/well of ^3^H-thymidine (Perkin Elmer, Boston, MA), and proliferation was measured by quantifying its incorporation through liquid scintillation counting after 18–22 h.

### T cell cytokine secretion assays

To assess T helper (Th) cell polarization or cytotoxic T lymphocyte (CTL) activation, we measured the secretion levels of cytokines produced by CD4^+^ or CD8^+^ T cells, respectively. For Th cell polarization, naive CD4^+^CD45RA^+^ T cells were stimulated using either immature dendritic cells (iDCs) or DCs that had been stimulated with MPLA/IFN-γ for 6 hours, 24 h, or 48 h. Co-cultures were established in 48-well tissue plates, with each well containing 1 × 10^6^ naive CD4^+^CD45RA^+^ T cells and 1 × 10^5^ corresponding allogeneic DCs (with varying degrees of maturation) as stimulators. RPMI 1640 culture medium supplemented with 10% human AB serum was used for these co-cultures. After 7 days of co-culture, the T cells were harvested, washed, re-plated at 2 × 10^6^ cells/well (96-well plate), and re-stimulated through their T cell receptor using T cell activation/expansion macrobeads (anti-CD2/CD3/CD28) from Miltenyi Biotec. Following a 24-hour incubation, the supernatants from the T cells were collected, and the levels of IL-4, IL-10, IL-17 A, TNF-α and IFN-γ were measured using the cytokine bead array method (CBA assay, BD biosciences) in accordance with the manufacturer’s protocol.

For experiments with CD8^+^ T cells, co-cultures were conducted in 48-well tissue plates, where each well contained 1 × 10^6^ CD8^+^ T cells obtained by immunomagnetic selection and 1 × 10^5^ DCs obtained with different stimulation conditions (iDCs and DCs exposed to maturation stimuli for 6 hours, 24 h, and 48 h). After 5 days, supernatants were directly collected from the DC: CD8^+^ T cell co-cultures and analyzed for the presence of IFN-γ, TNF-α, and IL-6 using the CBA assay (BD biosciences). The obtained results were analyzed using a FACSCalibur flow cytometer.

### Granzyme B expression analysis

To determine the intracellular expression of granzyme B, we performed 5 day co-cultures between variously matured DCs (either non treated or stimulated with MPLA and IFN-γ for 6 h, 24–48 h), and positively selected whole CD8^+^ T cells. On the final day of co-culture, the T cells were harvested, washed twice, and stained using APC-conjugated anti-CD8 mAb. Afterwards, the cells were washed, fixed and permeabilized with Fix/Perm Buffer (Biolegend, CA, USA). Subsequently, the permeabilized cells were stained with PE-conjugated anti-granzyme B mAb (Biolegend, CA, USA). The results were analyzed on a MACSQuant 10 flow cytometer (Milteny Biotec).

### Analysis of DC migration capacity

To evaluate the migratory capacity of DCs, we utilized the Transwell® system (from Corning, NY, USA) equipped with 8.0 μm polycarbonate filter inserts featuring an 8.0 μm pore size. Terminally differentiated iDCs, and DCs matured using various maturation times, were collected and subjected to two washes with DPBS. Subsequently, the cells were suspended in Cellgenix® DC GMP medium. A total volume of 100µL of the cell suspension was then added to the upper compartments of the transwell system. In the lower compartments, 500µL of medium supplemented with 200 ng/mL of chemokine CCL21 (Peprotech, London, UK) was placed. The entire plate was then incubated at 37 °C with 5% CO2 for a duration of 3 h. Afterward, the transwell inserts were carefully lifted, and the migrated cells from the lower compartments were collected. The flow cytometry technique was employed to quantify the number of migrated cells. A FACSCalibur flow cytometer (BD biosciences) was utilized for this purpose, and the cells were counted for 60 s, following previously described protocols [17].

### Analysis of Ag-specific CD8^+^ T cell responses

To obtain purified whole CD8 + T cells (purity > 90%), we employed positive immunomagnetic selection using CD8 microbeads from Miltenyi Biotech, Bergisch Gladbach, Germany. For the co-culture, 2 × 10^6^ cells of CD8^+^ T cells were sensitized using 2 × 10^5^ autologous iDCs or 2 × 10^5^ DCs stimulated with MPLA/IFN-γ for 6 hours or 24 h. Both the DCs and CD8^+^ T cells were from the same HLA-A2^+^ donor. The DCs were pulsed for 3 h with four melanoma-associated, HLA-A2-restricted antigen peptides (10 µg/mL of eachpeptide was used). The specific peptides used were gp100 (154–162, KTWGQYWQV), gp100 (209–217, ITDQVPFSV), tyrosinase (369–377, YMDGTMSQV), and melan-A (26–35, ELAGIGILTV), all obtained from Panatech, Heilbronn, Germany.

The co-culture was maintained for a period of 14 days. On day 0 and day 7, we supplemented the culture with 10 ng/mL of IL-7 and 50 U/mL of IL-2, both sourced from Peprotech, London, UK. On day 3 and day 10, we added the same concentrations of IL-7 and IL-2. On day 7, the T cells underwent re-stimulation using dendritic cells in the same manner as the initial stimulation. After 14 days, the T cells were harvested and re-plated at a density of 1 × 10^5^ cells on pre-coated IFN-γ ELISPOT strips from AID Autoimmun Diagnostika GmbH, Strassberg, Germany. These cells were re-stimulated with the T2 cell line (ATCC), serving as the stimulator cells (5 × 10^4^ cells per well), which were pre-pulsed with the same four melanoma-associated peptides as were used for the pulsation of DCs. Spot-forming colonies and their numbers were assessed using an ELISPOT reader from AID Autoimmun Diagnostika, Strassberg, Germany.

### Statistical analysis

The statistical analysis was conducted utilizing Graphpad Prism® software version 6.07 for Windows (Graphpad, San Diego, CA, USA). To determine the statistical significance between individual pairs, Student’s unpaired t-test was employed. A *p*-value of less than 0.05 was considered to indicate statistical significance. The assumption of normality in the distribution of data was verified by conducting the Shapiro-Wilk normality test.

## Results

### Short-term maturation of DCs leads to major increase in cytokine production upon CD40-Ligand stimulation

**Fig. 1:**
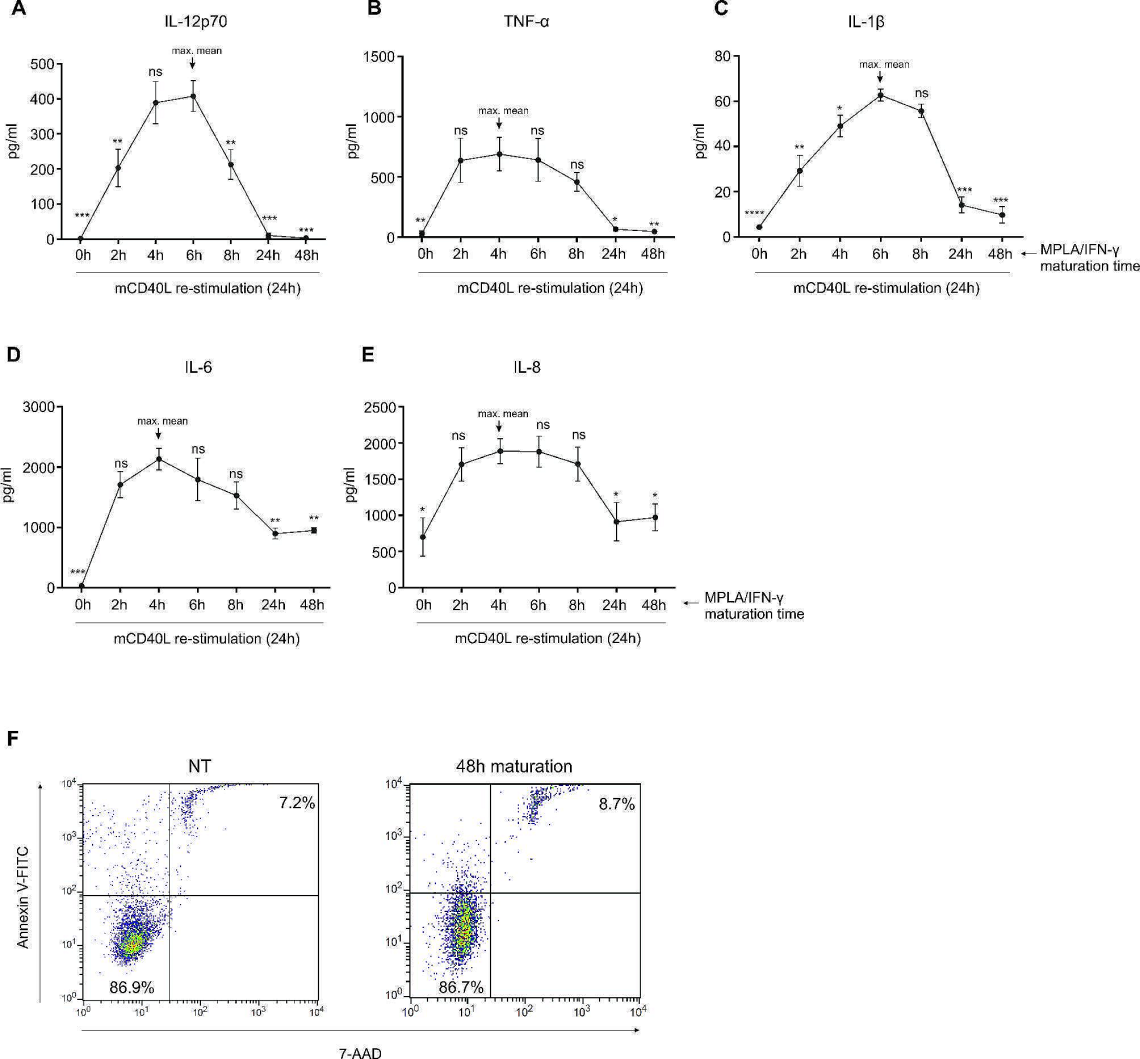
Short-term maturation leads to substantially enhanced responsiveness of DCs to CD40 Ligand re-stimulation. Dendritic cells were generated from peripheral blood monocytes, as described in Materials & Methods. They were exposed to a time titration (2 h, 4 h, 6 h, 8 h, 24 h and 48 h) of maturation signals composed of MPLA and IFN-γ. Afterwards, the cells were washed and re-stimulated using multiple CD40 Ligand for 24 h and cell culture supernatants were analyzed for the presence of **(A)** IL-12p70, **(B)** TNF-α, **(C)** IL-1β, **(D)** IL-6 and **(E)** IL-8. Data represent mean ± SD of five independent experiments. **(F)** Viability of either non-treated (NT) or DCs matured for 48 h (48 h maturation) was determined, as described in Materials & Methods. Statistical significance was calculated for comparison of pairs between the sample with maximum mean (depicted in the figure) with individual samples, as depicted. The significance in difference was calculated using Student’s unpaired t test. A *p* value of less than 0.05 was considered statistically significant (ns – non-significant; * - *p* < 0.05; ** - *p* < 0.01; *** - *p* < 0.001; **** - *p* < 0.0001)

Dendritic cells were differentiated from peripheral blood monocytes, as described in the Materials & Methods section. Immature DCs were harvested on day 6 and characterized as CD1a^high^, CD14^low^, CD209^high^ (data not shown). To determine the importance of temporal exposure of DCs to maturation stimuli, we matured DCs using MPLA/IFN-γ for the following time periods: 0 h, 2 h, 4 h, 6 h, 8 h, 24 h and 48 h. The protocol for maturation was set in such a way, that all samples could be harvested simultaneously. After harvest, the DCs were washed extensively and re-stimulated with CD40-Ligand multimer kit for 24 h. After CD40-Ligand stimulation, the culture supernatants were collected and analyzed for the presence of IL-12p70, TNF-α, IL-1β, IL-6 and IL-8 (Fig. 1A, B, C, D, E and F, respectively). Interestingly, DCs that were matured for 24 h or longer, display a steep drop in secretion of most of the analyzed cytokines, after CD40-Ligand re-stimulation. This is particularly evident for IL-12p70 (Fig. 1A), as well as for TNF-α (Fig. 1B) and IL-1β (Fig. 1C). CD40Ligand induced peak cytokine production in DCs matured for 4–6 h. The overall reduction in producing capacity (over 100-fold) was most extensive for IL-12p70, where 48 h maturation of DCs resulted in almost null capacity to produce IL-12p70 after re-stimulation.

### Brief exposure to maturation signals allows for co-stimulatory molecule expression on DCs

**Fig. 2:**
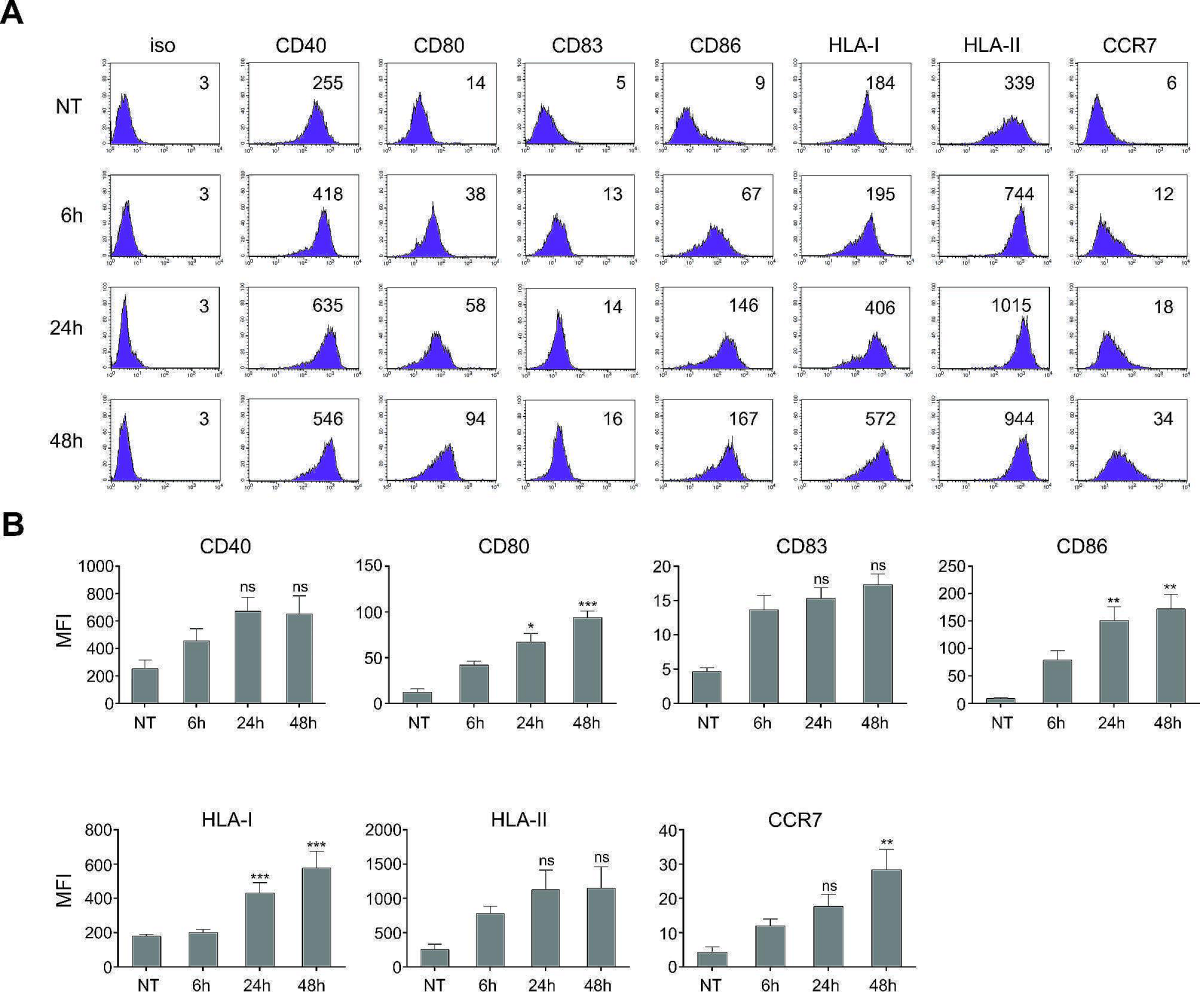
Short-term maturation allows for marked expression of co-stimulatory molecules. Dendritic cells were generated from monocytes isolated from freshly prepared human buffy coats, as described in Materials & Methods. They were then either left untreated (NT – immature DCs) or were stimulated with a combination of MPLA and IFN-γ for a period of 6 h, 24–48 h. Afterwards, the cells were analyzed for surface expression of broad phenotypic markers associated with DC maturation. **(A)** The numbers in histograms represent the mean fluorescence intensity values (geometric mean). Shown is one representative experiment out of three performed. **(B)** Bar graph representation of all three independent experiments. Results are presented as mean ± SD (MFI – mean fluorescence intensity). Data were analyzed on a FACSCalibur system using CellQuest software version 3.3 (BD biosciences). The statistical significance between individual pairs (6 h group vs. 24 h group or 48 h group) was calculated using Student’s unpaired t test. A *p* value of less than 0.05 was considered statistically significant. (ns – non significant; * - *p* < 0.05; ** - *p* < 0.01; *** - *p* < 0.001) NT – non-treated DCs; 6 h, 24 h, 48 h - DCs matured for 6 h, 24–48 h, respectively

Immature DCs differentiated from peripheral blood monocytes and harvested on day 6, as described in Materials & Methods. They were then either left un-stimulated or stimulated with MPLA/IFN-γ for 6 h, 24–48 h. Afterwards, the cells were washed, replated and restimulated with CD40-Ligand for 24 h. The cells were then harvested and stained for broad phenotypic analysis (Fig. 2). In general, 6 h exposure to initial maturation stimuli was sufficient for DCs to express evident levels of CD40, CD80 and CD86 co-stimulatory molecules. The expression of lymph node-homing receptor CCR7, as well as HLA class II molecules were also significantly induced. As expected, the longer exposures to initial maturation signals resulted in more extensive up-regulation of nearly all maturation-associated markers (approx. 2–3 fold in general).

### DCs matured for shorter periods display superior allostimulatory capacity

**Fig. 3:**
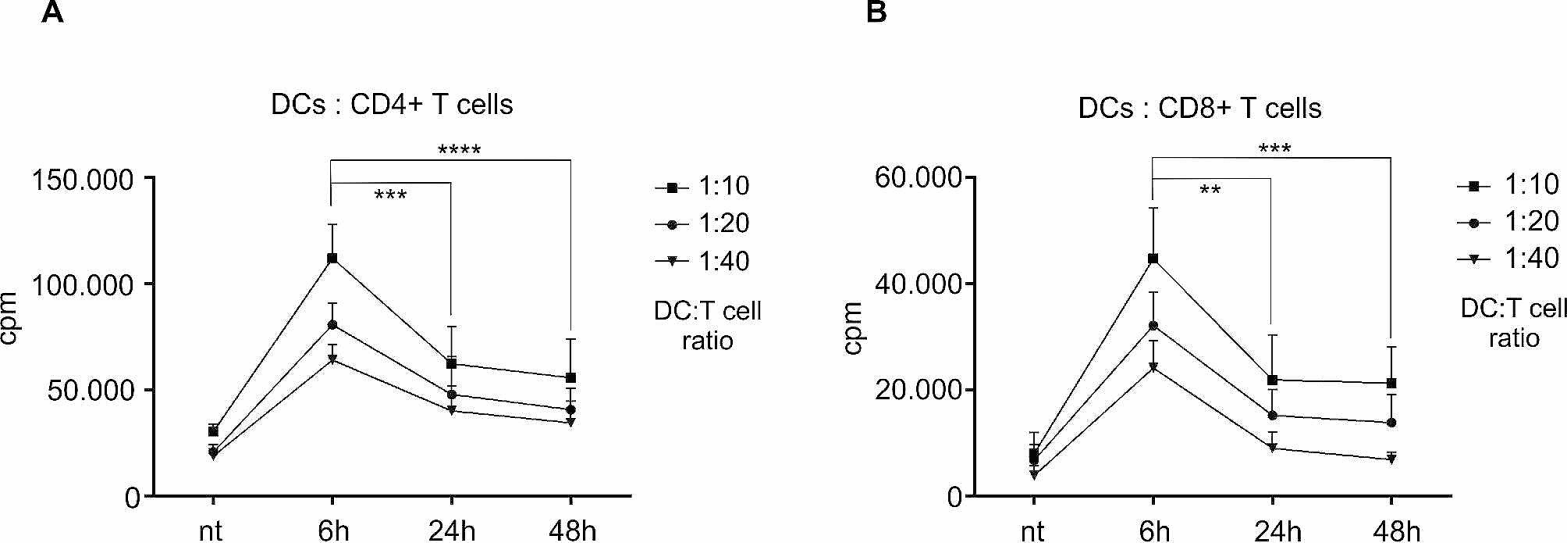
Briefly-matured DCs possess superior allo-stimulatory capacity. Dendritic cells matured for various time periods were evaluated for their capacity to stimulate the proliferation of allogeneic T cells. **(A)** The capacity of DCs to induce proliferation of allogeneic, whole CD4^+^ T cells was assessed. DC: T cell co-cultures were set-up in 96 wells in a 1:10, 1:20 or 1:40 ratio in 200 µl of RPMI + 10% human AB serum. On day 4, the wells were pulsed with 1µCi of tritium-labeled thymidine. Proliferation was measured on day 5 by liquid scintillation counting. **(B)** The capacity of DCs to induce proliferation of allogeneic, whole CD8^+^ T cells was determined in identical manner. The results are shown as mean ± SD of counts per minute (cpm) of 6 independent experiments. Statistical significance between individual pairs (6 h group vs. 24–48 h group) was calculated using Student’s unpaired t test. A *p* value of less than 0.05 was considered statistically significant (** - *p* < 0.01; *** - *p* < 0.001; **** - *p* < 0.0001). nt – non-treated DCs; 6 h, 24 h, 48 h - DCs matured for 6 h, 24–48 h, respectively

To assess the capacity of DCs matured for shorter or longer time periods to stimulate proliferation of T cells, we used differentially stimulated DCs in a mixed lymphocyte reaction co-culture. For stimulators, we used immature DCs or DCs mature with MPLA/IFN-γ for 6 h, 24–48 h. Co-cultures were performed in 96 flat bottom wells with a DC: T cell ratio of 1:10 (2 × 10^4^ DCs and 2 × 10^5^ T cells per well). Responder cells consisted of immunomagnetically selected whole CD4^+^ T cells or whole CD8^+^ T cells. Cell proliferation was measured on day 5 by liquid scintillation counting (Fig. 3A and B). As expected, immature DCs possessed poor allo-stimulatory capacity, compared to mature DCs. However, DCs matured for 6 h possessed approximately twice the capacity of 24 h and 48 h-matured DCs to induce proliferation of both CD4^+^ (cpm counts 108.908 ± 9224, 62.491 ± 16.192 and 55.824 ± 16.652 for 6 h, 24 h and 48 h maturation, respectively) and CD8^+^ T cells (cpm counts 41.464 ± 7019, 21.941 ± 7785 and 21.305 ± 6241 for 6 h, 24 h and 48 h maturation, respectively).

### DCs matured for shorter periods are far more efficient at inducing type 1 T cell responses

**Fig. 4:**
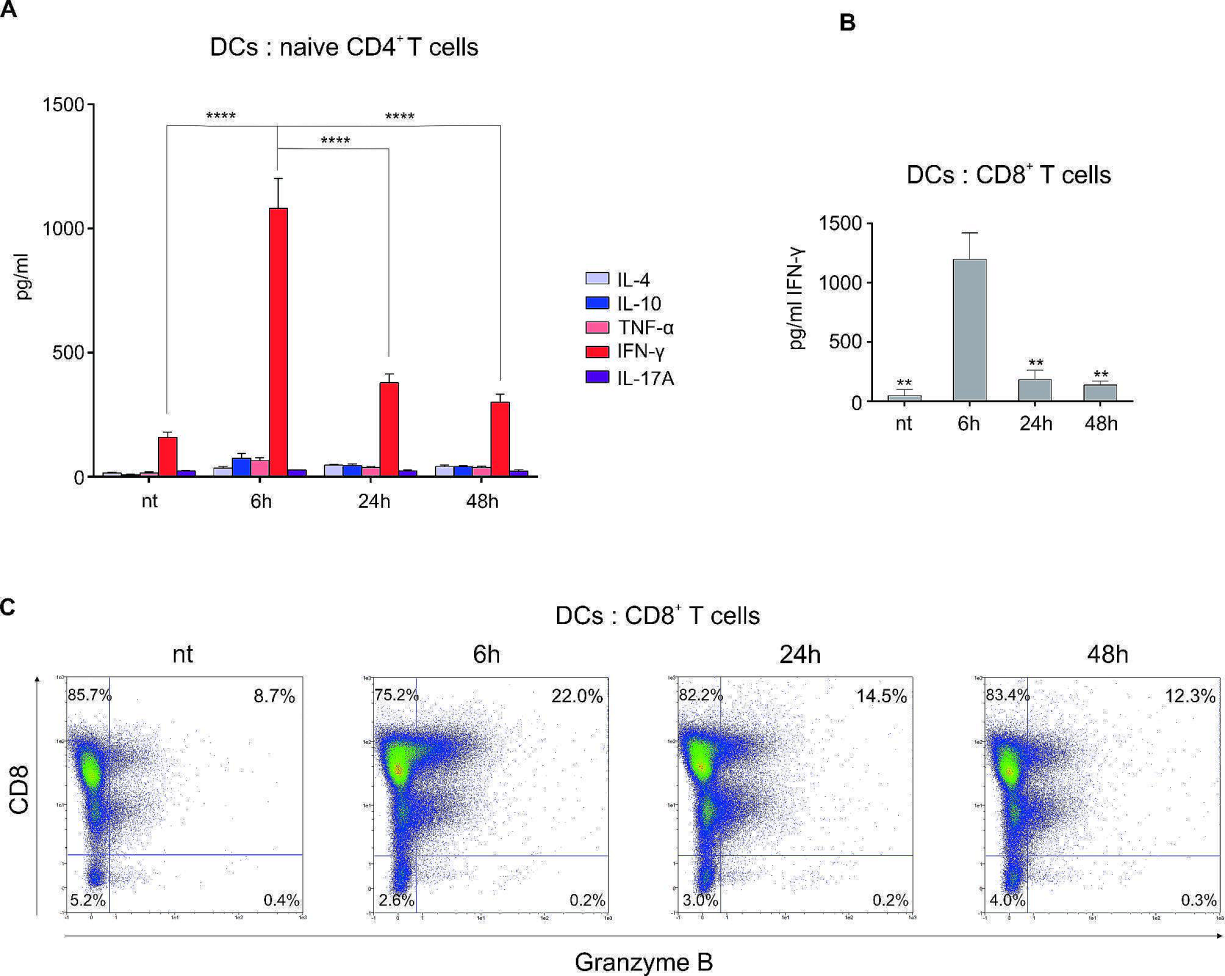
Short-term maturation endows DCs with superior induction of type 1 T cell responses. Dendritic cells were generated as described in Materials & Methods. **(A)** The capacity of DCs to induce polarization of CD4^+^ T cells was determined by performing co-cultures of differentially-matured DCs with allogeneic, naive CD4^+^CD45RA^+^ T cells (48 well tissue culture plates, DC: T cell ratio 1:10). The co-cultures were executed in RPMI medium supplemented with 10% human AB serum. After 7 days, the T cells were collected and re-stimulated via TCR using anti-CD2/CD3/CD28 macrobeads. The levels of IL-4, IL-10, TNF-α, IFN-γ and IL-17 A were determined in cell culture supernatants after 24 h of re-stimulation. **(B)** The capacity of DCs to induce activation of CD8^+^ T cells was determined in co-cultures of variously matured DCs with whole CD8^+^ T cells. After 5 days, the co-culture supernatants were analyzed for the presence of IFN-γ. Data are presented as mean ± SD of three independent experiments. Statistical significance between pairs of 6 h-matured DCs and individual samples, as depicted in the Figure. **(C)** The capacity of variously matured DCs to induce CD8^+^ T cell activation was determined by intracellular granzyme B expression. Co-cultures of DCs and whole CD8^+^ T cells were maintained for 5 days. Afterwards, the T cells were collected and stained intracellularly, as described in Materials & Methods. Shown is one representative experiment from three independent experiments performed. A *p* value of less than 0.05 was considered statistically significant (ns – non-significant; ** - *p* < 0.01; **** - *p* < 0.0001). nt – non treated DCs; 6 h, 24 h, 48 h – DCs matured for 6 h, 24–48 h, respectively

Subsequently, we wanted to study the functional capacity of DCs matured for differential time periods, to induce T helper cell polarization. We therefore cultured immature DCs, or DCs matured with MPLA/IFNγ for various time periods, with immunomagnetically isolated naive, CD4^+^CD45RA^+^ T cells, as described in the Materials & Methods section. The co-cultures were performed in 48-well tissue culture plates. In all instances we performed the co-cultures using RPMI supplemented with 10% human AB serum. After 7 days, the capacity of induced T helper cells to produce various cytokines was determined by re-stimulation of T cells via their TCR. Levels of IL-4, IL-10, TNF-α, IFN-γ and IL-17 A were determined in final culture supernatants (Fig. 4A). In general, DCs matured with MPLA/IFN-γ drove the polarization of type 1 T helper responses, reflected as predominant presence of IFN-γ in culture supernatants after TCR-mediated restimulation. Interestingly, DCs exposed to short term maturation (6 h) were much more efficient than long term-matured DCs at driving Th1 polarization, with almost 3-fold higher IFN-γ levels detected. We also evaluated the capacity of variously matured DCs to induce CTL activation, by measuring IFN-γ levels and their capacity to induce intracellular granzyme B exppression after 5 day co-culture with whole CD8^+^ T cells (Fig. 4B and C). To an even greater extent, the short term maturation of DCs proved much more efficient than long term in CD8^+^ T cell activation, inducing over 8-fold greater IFN-γ production. The results were also confirmed by superior capacity of briefly-matured DCs to induce approximately 50% greater granzyme B competence in CD8^+^ T cells (Fig. 4C).

### Brief maturation of DCs is sufficient for effective CCR7-directed DC migration

**Fig. 5:**
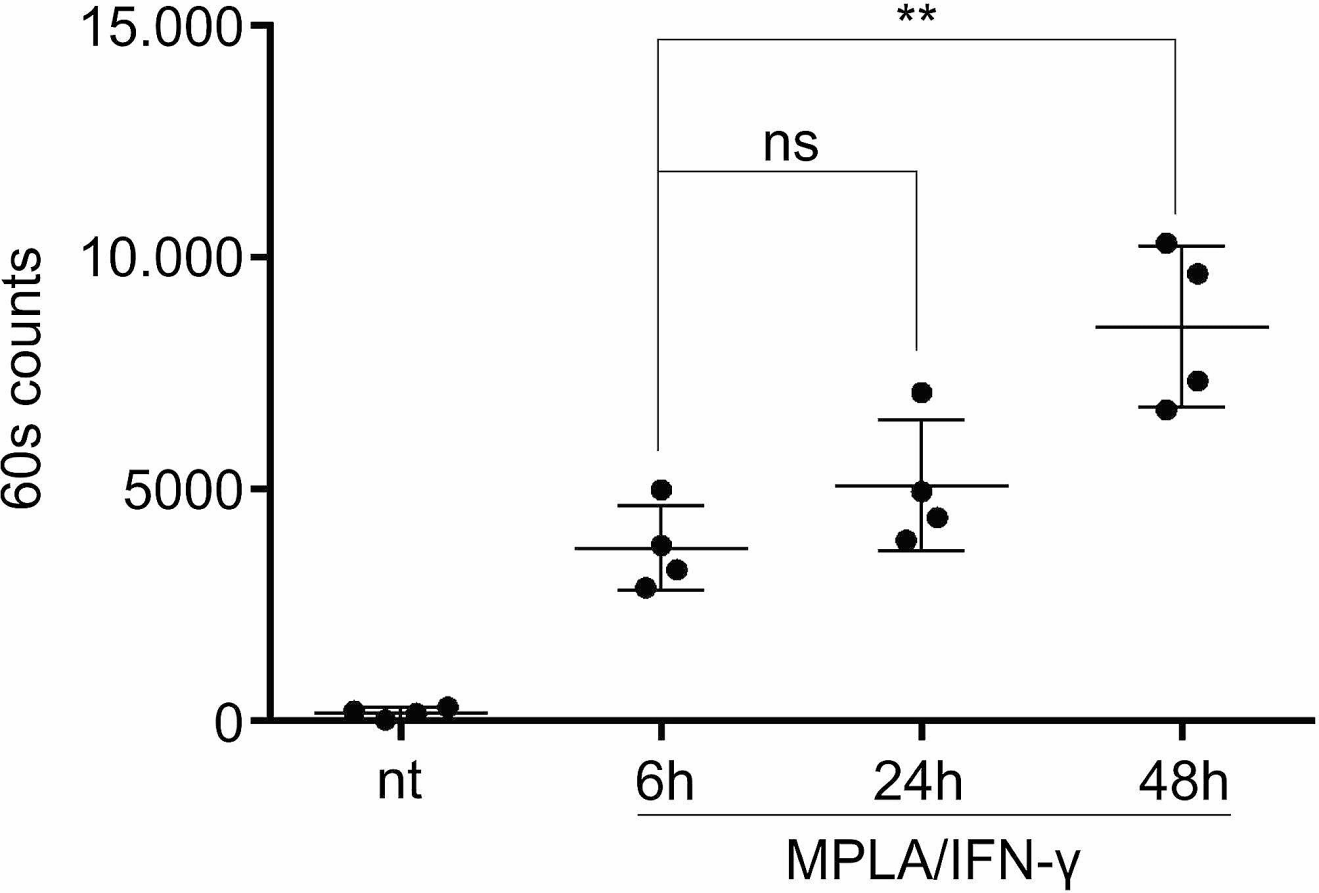
Short-term maturation of DCs is sufficient for induction of CCR7-mediated migratory capacity. We differentiated DCs from peripheral blood monocytes, as described in Materials & Methods. The CCL21-directed homing capacity of DCs, matured for various time periods using MPLA and IFN-γ, was evaluated using a transwell migration assay. Non-treated, immature DCs were used for negative control. Lower chambers of the transwell contained 200 ng/ml of recombinant CCL21 protein and the assay was performed for 3 h at 37 °C and 5% CO_2_. We estimated the number of migrated DCs by counting the migrated cells from the lower chamber by flow cytometry using 60 s counts. The results represent mean ± SD of four independent experiments. The dots represent values of individual samples. Statistical significance between depicted pairs was calculated using Student’s unpaired t test. A *p* value of less than 0.05 was considered statistically significant (ns – non-significant; ** - *p* < 0.01). nt – non treated DCs; 6 h, 24 h, 48 h – DCs matured for 6 h, 24–48 h, respectively

The CCR-7-mediated migratory capacity of DCs is considered important in context of DC vaccines, as effective migration of DCs to secondary lymph nodes is crucial for subsequent contact with responding T cells. To determine the CCL21-directed homing capacity of differentially matured DCs, we performed a transwell assay, as described in Materials & Methods section. We used unstimulated, immature DCs as controls, as well as DCs stimulated with MPLA/IFN-γ for 6 h, 24–48 h. We left the migration assay take place for 3 h in a humidified incubator and afterwards collected the cells from lower chambers and counted them using 60s counts (Fig. 5). There was an evident increase in the number of migrated mature DCs in comparison to immature DCs, as expected. Although briefly-matured DCs migrated efficiently, their CCR7-directed migratory capacity was lower than with DCs matured for longer periods, which was particularly evident when compared with 48 h-matured DCs.

### Briefly-matured DCs have a superior capacity to induce melanoma-specific CD8^+^ T cell responses

**Fig. 6:**
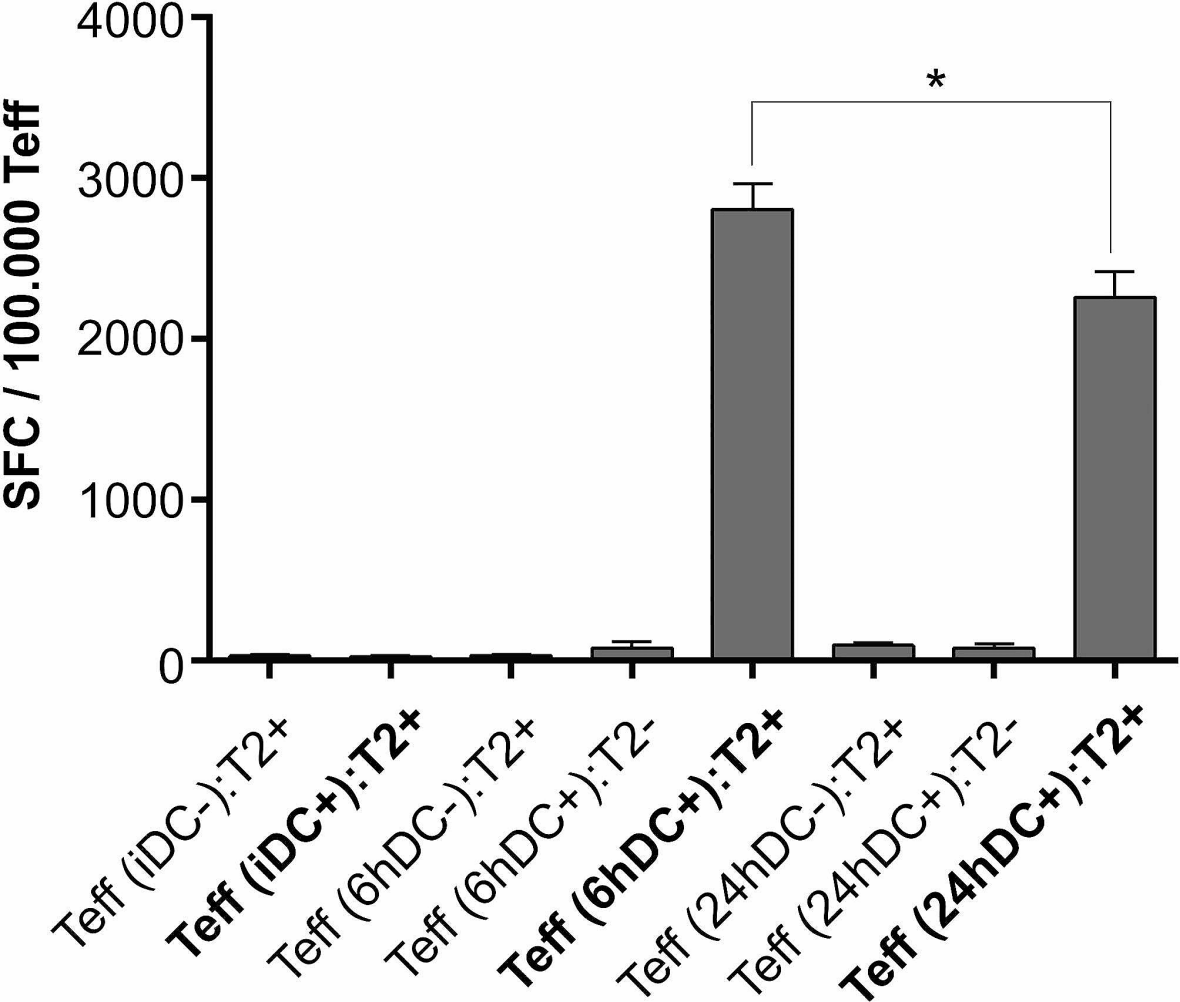
Dendritic cells exposed to maturation stimuli for shorter time periods display increased capability to induce melanoma-specific CTL responses. We sensitized autologous, whole CD8^+^ T cells by applying two rounds of stimulation with either immature DCs (iDC), or DCs matured for 6 h (6hDC) or 24 h (24hDC). Prior to this, the DCs were pulsed (DC+) with melanoma-associated peptides gp100 (154–162), gp100 (209–217), tyrosinase (369–377), and melan-A (26–35), or left unpulsed for controls (DC-). After 14 days of stimulation in DC: T cell co-culture, the T cells were harvested and re-stimulated with T2 cells as stimulators (precedently pulsed with identical melanoma peptides – T2+, or left unpulsed – T2-). Re-stimulation proceeded on pre-coated, IFN-γ ELISPOT strips for 24 h. The number of spot-forming colonies was determined and analyzed using ELISPOT reader. The data are shown as mean ± SEM of duplicates of three independent experiments. Statistical significance between relevant, individual pairs was calculated using Student’s unpaired t test. A *p* value of less than 0.05 was considered statistically significant (* - *p* < 0.05)

We wanted to study whether changes in temporal exposure of DCs to maturation stimuli can also be detected in prolonged experimental settings, such as induction of Ag-specific T cell responses. For this purpose we cultured monocyte-derived DCs matured for 6–24 h with whole, HLA-A2^+^ autologous CD8^+^ T cells. The DCs were pulsed with four melanoma-associated peptides prior to co-culture with T cells. After 14 days, T cell responses against Ags gp100 (154–162), gp100 (209–217), tyrosinase (369–377), and melan-A (26–35), were determined via counting the number of spot-forming colonies (SFCs) using the ELISPOT method (Fig. 6). While immature DCs had practically no capacity to induce melanoma-specific CTLs, mature DCs caused significant induction of SFCs. In this regard, short term-matured DCs were again superior compared to long term maturation protocol, inducing significantly greater number of SFCs, with 29.5 ± 3.5, 2809 ± 127 and 2260 ± 129 SFCs for iDCs, 6 h-matured DCs and 24 h-matured DCs, respectively.

### The significance of temporal exposure for IL-12p70 production is not limited to TLR4

**Fig. 7:**
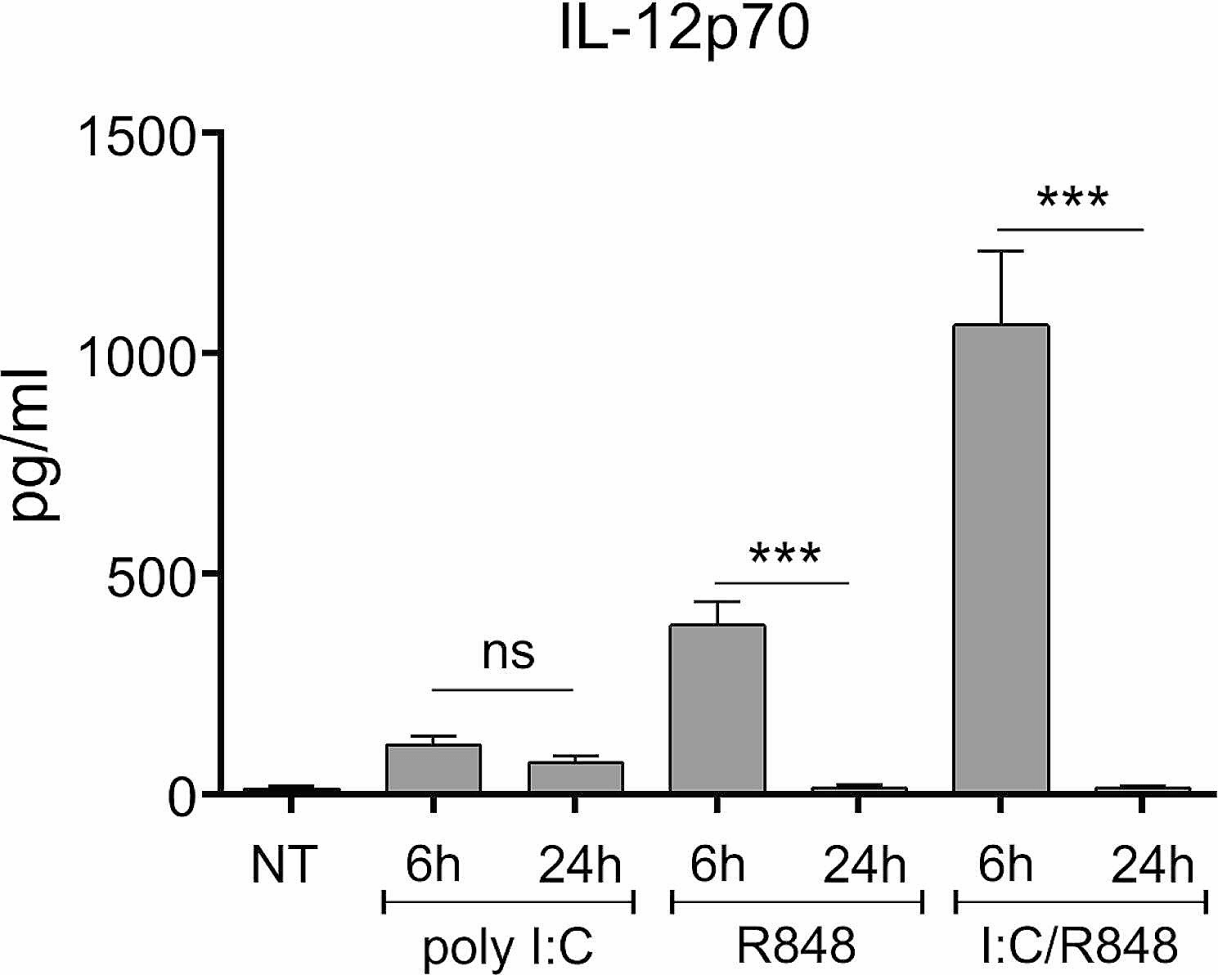
The effect of temporal exposure on type 1 DC maturation applies to various TLR pathways. We generated DCs from human peripheral blood monocytes, as described in Materials & Methods. After differentiation, the DCs were exposed to TLR3 and TLR8 agonists (activation pathways most frequently used for type 1 DC maturation, in addition to TLR4 agonists), or the combination of both, for a time period of 6–24 h. After maturational stimuli, the cells were washed and re-stimulated using multiple CD40 Ligand for 24 h. At the end of re-stimulation period, the cell culture supernatants were analyzed for the presence of IL-12p70. Data are presented as mean ± SD of three independent experiments. Statistically, we compared individual pairs of samples of DCs stimulated for 6–24 h, independently for each stimuli, as depicted in the figure. The significance was calculated using Student’s unpaired t test. A *p* value of less than 0.05 was considered statistically significant (ns – non-significant; * - *p* < 0.05; ** - *p* < 0.01; *** - *p* < 0.001). nt – non treated DCs; 6 h, 24 h – DCs matured for 6–24 h, respectively

Our “golden standard” for type 1 DC maturation in this study has been the well documented double signal consisting of TLR4 ligand MPLA in combination with IFN-γ. We wanted to study whether the observed substantial effect of differential temporal exposure is perhaps limited to DC activation via TLR4 pathway. For this reason we performed additional experiments where brief- (6 h) and long-term (24 h) maturation in DCs was induced by a TLR3 ligand (poly I: C), TLR8 ligand (R848 – resiquimod) or the combination of both, the latter serving as an alternative double signal. Similarly to our previous experiments, the DCs exposed to maturation stimuli were washed, and re-stimulated via the CD40-CD40L pathway for 24 h. The cell culture supernatants were afterwards analyzed for the presence of IL-12p70 (Fig. 7). The 24 h maturation resulted in substantially reduced capacity of DCs to produce IL-12p70, compared to 6 h maturation. The most extensive effects of temporal exposure were seen in case of stimulation using double TLR agonist singnal I: C/R848. Identically as in the case of MPLA/IFN-γ, the capacity of DCs to produce IL-12p70 after 24 h was almost completely diminished.

## Discussion

Maturation of DCs is one of the most decisive features of their functional biology. It plays a central role in their operative positioning within the complex network of our immune system, while on the other hand, its understanding grants us the tools to develop novel and optimized DC-based therapies [18]. In the present manuscript, we present a very seldom addressed issue, which is the importance of temporal exposure to maturation signals in regard to DCs’ subsequent qualitative functional features. In light of important representation of DCs in the field of anti-cancer cellular vaccine preparation, we focused on the combination of TLR-4 agonist MPLA and IFN-γ as our primary maturation protocol, with well documented capacity to induce type 1 immune responses, the latter being central to anti-cancer immunity [19, 20]. Firstly, we evaluated our thesis for the importance of temporal exposure by time titration (0 –48 h) of DC maturation and their capacity to produce IL-12p70 and other innate cytokines upon re-stimulation via the CD40-CD40 Ligand pathway. We primarily focused on cytokine secretion in response to CD40 ligation since it represents the closest approximation to DCs’ bidirectional interaction with responding T cells, as well as it being more informative than cytokine analysis after primary maturation [21]. We observed that production of all inflammatory cytokines followed a similar and apparent pattern, whereby the peak of DCs’ production capacity was reached after 4–6 h exposure to primary maturation stimuli (MPLA/IFN-γ). After this time, production capacity fell sharply, particularly in the case of IL-12p70, the major type 1 polarizing cytokine. It is important to note that in cases, where primary maturation lasted 24–48 h, the capacity of DCs to produce IL-12p70 almost became absent. A similar, although a less extreme decline was seen for TNF-α and IL-1β, while the production of IL-6 and IL-8 was somewhat less affected. As seen in Fig. 1, the window for optimal signal 3 (soluble factors produced by DCs) delivery to T cells from DCs is situated around 6 h of primary exposure of the latter to maturation signals and was the reason for choosing the 6 h maturation period for our brief maturation protocol in subsequent experiments.

In order to breach the threshold for T cell activation, DCs must deliver a sufficient amount of co-stimulatory signals after the formation of the immunological synapse [22]. In vitro, this is most often achieved by treating DC cultures with various immunogenic signals, however most measurements in the past have been made after 1–2 days of stimulation, after which time DCs express significant up-regulation of various co-stimulatory molecules. In our case, DCs matured for 6 h did not show the same level of co-stimulatory molecule expression as those with standard maturation times, however, 6 h was still sufficient for DCs to achieve significant expression of CD80, CD86, as well as CCR7 and HLA class II molecules. There is no consensus on how extensive, compared to immature DCs, the presence of surface co-stimulatory molecules should be for efficient T cell activation. For this reason, we wanted to evaluate how efficient differentially matured DCs are in their allo-stimulatory capacity. Surprisingly, although in general there was an approximate 2-fold lower presence of co-stimulatory molecules on briefly matured DCs vs. those matured for longer, briefly matured DCs displayed an outstanding allo-stimulatory capacity, 2-fold greater than DCs matured for 24–48 h. This was the case for both CD4^+^ and CD8^+^ T cells, as responders. This is an interesting and rather unexpected observation, since T cell activation/proliferation is more dependent on successful delivery of signal 2 (co-stimulation) than signal 3 (cytokines) [23]. We see two possible explanations for this, one being that once DC activation threshold is reached, T cells can deliver additional co-stimulation via CD40 ligation to achieve sufficient DC maturation and subsequently, efficient DC-mediated co-stimulation during co-culture. The other is that co-stimulatory molecule expression achieved by our brief maturation protocol could be more than sufficient, in other words, have already reached the required plateau for T cell activation. Moreover, brief maturation of DCs could contribute to lesser exhaustion of DCs, leading to improved “fitness” and capability to stimulate T cells for longer before the emergence of negative feedback signals, such as expression of PD-L1 or even decrease in migratory capacity, which could attenuate T cell activation [24–26].

Even greater differences than with allo-stimulatory capacity, have been seen between briefly and normally matured DCs in respect to their type 1 polarization capacity. T cell populations generated from naïve CD4^+^ T cells during 7-day stimulation with DCs matured for 6 h, were capable of producing approximately 3 times as much IFN-γ, compared to those stimulated with 24 h- or 48 h-matured DCs. Similar or even greater type 1 polarization capacity was observed with CD8^+^ T cell activation. We believe the much stronger capacity of briefly-matured DCs to produce IL-12 after re-stimulation is one of the main reasons for this observation. The reason this effect is even more pronounced in our CD8^+^ T cell assay is most likely the greater dependence of CD8^+^ T cells on helper cytokines [27]. In this way, below-the-threshold signal 3 delivery from DCs could greatly thwart CD8^+^ T cell activation, resulting in very low IFN-γ levels these cells could produce in our experiment, when stimulated with 24 h- or 48 h-matured DCs (Fig. 4). On the other hand, CD8^+^ T cell response would be significantly induced in case of sufficient or even copious presence of signal 3. This was additionally confirmed by measuring CD8^+^ T cell activation capacity via granzyme B expression, which was most efficiently induced in T cells stimulated by 6 h-matured DCs (Fig. 4C).

We also confirmed the importance of temporal aspect of DC maturational stimulation in a more complex and prolonged experimental settings, namely the induction of Ag-specific autologous CTLs. We assessed the number of SFCs using ELISPOT after a 14 day co-culture between autologous CD8^+^ T cells and DCs pulsed with four melanoma-associated peptides. A significantly greater number of Ag-specific CTLs was observed from co-cultures, where briefly matured DCs were used as stimulators, compared to those with 24 h-matured DCs. Although there were clear differences in our ELISPOT experiments between differentially matured DCs, it is clear these differences were not as extensive as those seen e.g. in Figs. 3 and 4. While we do not have a complete explanation for this, we speculate the differences can come from autologous vs. allogeneic experimental designs. In this manner, the number of Ag-specific precursors in the ELISPOT assay would be much lower than in our allogeneic settings, where exponential activation of much greater number of responding T cells could contribute to final increase in observed differences between samples.

Interestingly, although the brief maturation protocol also allowed for efficient migration of DCs in a CCR7-directed manner (evidently greater compared to immature DCs), the migration capacity was at the same time the only functional aspect we observed that was more favorably expressed by longer maturation periods. This was particularly evident for 48 h maturation protocol. There is little doubt this increase in migration capacity can be associated with greater expression of CCR7 on DCs matured using longer maturation periods (Fig. 2). However, the in vivo relevance of this should be further determined in the future, particularly in light of various routes of administration (e.g. local vs. systemic).

In the past, it has been well established that for efficient type 1 polarization and high production of IL-12, the DC maturation requires two signals [28]. For example, these can represent a combination of IFN-γ and T cell co-stimulation. However, the two signal requirement is not limited to these factors, as they can be readily replaced with e.g. lipopolysaccharide, a TLR4 ligand. Nevertheless, we wanted to extend the findings in our study and see if combinations of other TLR pathways also play a role in the time-dependent mechanisms of DC maturation we have observed with MPLA/IFN-γ stimulation. We therefore designed our experiment where DCs were stimulated with poly I: C, R848 or a combination of both for short (6 h) or longer (24 h) time period. The IL-12p70 producing capacity of DCs was then analyzed upon CD40 Ligand re-stimulation (Fig. 7). The negative effect of long-term maturation was very much evident, with the production of IL-12p70 being practically absent after longer initial maturation, while short-term maturation caused a high spike of IL-12p70 production capacity.

The molecular signaling mechanisms involved in DC maturation are intricate and involve a myriad of pathways and molecules. When DCs are exposed to maturation stimuli such as TLR agonists and cytokines, a cascade of signaling events is triggered. For example, TLR4 activation by MPLA engages the MyD88-dependent pathway, leading to the activation of NF-κB and the production of pro-inflammatory cytokines [12]. Concurrently, IFN-γ signaling activates the JAK-STAT pathway, particularly STAT1, which is crucial for the transcription of genes involved in type 1 immune responses [29]. These signaling pathways converge to enhance the expression of co-stimulatory molecules and the secretion of cytokines like IL-12, which are pivotal for the activation and polarization of T cells.

The temporal dynamics of these signaling events are critical. Short-term exposure to maturation signals might preferentially activate early-response genes and pathways that enhance immediate DC function and cytokine production. In contrast, prolonged exposure could lead to feedback inhibition mechanisms, such as the induction of suppressive molecules like PD-L1, which dampen DC function [30]. This temporal regulation ensures that DCs can rapidly respond to danger signals but also avoid over-activation and potential exhaustion, which would impair their ability to sustain an effective immune response.

The first clinical use of DC-based vaccine has now been almost 30 years ago. Nevertheless, we are still to arrive at a point of understanding how to best take advantage of their potential. In the past, the major aspect of tackling this issue was by discovering novel mechanisms in terms of combinatorial signaling via various PRR and cytokine receptors, which has led to important and synergistic improvements in this context over the years. In this context, we have defined in our previous study a novel maturation cocktail, based on priming of DCs via TLR3, followed by stimulation via TLR8 and selected inflammatory cytokines, which results in greatly optimized type 1 polarization capacity of DCs, compared to previously established methods [15]. The results in our current study show this issue can be tackled from a previously unaddressed and clearly a key aspect of how to further advance the efficacy of DCs to induce type 1-polarized immune response, namely the optimal temporal exposure to maturation stimuli. One could argue that in certain cases of Ag pulsation, such as using whole tumor lysates or mRNA transduction, the DCs could need more than 6 h for complete Ag processing and loading. While this is a reasonable speculation, it is also likely that the completion of Ag processing on the cellular level would very likely continue after vaccine administration in vivo.

In conclusion, the findings presented in this manuscript offer valuable insights into the optimal exposure of DCs to maturation signals, with significant implications for clinical practice. Namely, the study highlights the importance of maturation timing, particularly in the context of inducing type 1 immunity, crucial for anti-cancer immune responses. By pinpointing the ideal duration of exposure to maturation signals, researchers can design more efficient and targeted vaccination protocols, ultimately bolstering the effectiveness of DC vaccines. This can also allow for tailoring of treatment regimens to optimize immune activation and tumor targeting in specific patient populations. The value of DC maturation quality has already been proven in the clinic, and this could have further implication in current era of immune checkpoint inhibitors that opened a new alleyway for DC vaccines. Importantly, our results can be easily implemented in future DC manufacturing protocols, since they are based on a straightforward premise, which can be smoothly incorporated in various standard operating protocols intended for clinical trials. In this manner, the quest for designing novel and improved DC manufacturing protocols is still very much a current topic.

### Electronic supplementary material

Below is the link to the electronic supplementary material.

Supplementary Material 1

## Data Availability

N/A.

## References

[1] Collin M, Bigley V (2018). Human dendritic cell subsets: an update. Immunology.

[2] Cabeza-Cabrerizo M, Cardoso A, Minutti CM (2021). Pereira Da Costa, C. Reis E Sousa, dendritic cells revisited. Annu Rev Immunol.

[3] Anguille S, Smits EL, Lion E, van Tendeloo VF, Berneman ZN (2014). Clinical use of dendritic cells for cancer therapy. Lancet Oncol.

[4] Palucka K, Ueno H, Fay J, Banchereau J (2011). Dendritic cells and immunity against cancer. J Intern Med.

[5] Butterfield LH (2013). Dendritic cells in cancer immunotherapy clinical trials: are we making progress?. Front Immunol.

[6] Harari A, Graciotti M, Bassani-Sternberg M, Kandalaft LE (2020). Antitumour dendritic cell vaccination in a priming and boosting approach. Nat Rev Drug Discov.

[7] Banchereau J, Briere F, Caux C, Davoust J, Lebecque S, Liu YJ, Pulendran B, Palucka K (2000). Immunobiology of dendritic cells. Annu Rev Immunol.

[8] Akira S (2009). Pathogen recognition by innate immunity and its signaling. Proc Jpn Acad Ser B Phys Biol Sci.

[9] Lutz MB (2016). Induction of CD4(+) Regulatory and polarized Effector/helper T cells by dendritic cells. Immune Netw.

[10] Schuurhuis DH, Laban S, Toes RE, Ricciardi-Castagnoli P, Kleijmeer MJ, van der Voort EI, Rea D, Offringa R, Geuze HJ, Melief CJ, Ossendorp F (2000). Immature dendritic cells acquire CD8(+) cytotoxic T lymphocyte priming capacity upon activation by T helper cell-independent or -dependent stimuli. J Exp Med.

[11] de Vries IJM, Lesterhuis WJ, Scharenborg NM, Engelen LPH, Ruiter DJ, Gerritsen M-JP, Croockewit S, Britten CM, Torensma R, Adema GJ, Figdor CG, Punt CJA (2003). Maturation of dendritic cells is a prerequisite for inducing Immune responses in Advanced Melanoma patients. Clin Cancer Res.

[12] Dalod M, Chelbi R, Malissen B, Lawrence T (2014). Dendritic cell maturation: functional specialization through signaling specificity and transcriptional programming. EMBO J.

[13] Kono M, Nakamura Y, Suda T, Uchijima M, Tsujimura K, Nagata T, Giermasz AS, Kalinski P, Nakamura H, Chida K (2012). Enhancement of protective immunity against intracellular bacteria using type-1 polarized dendritic cell (DC) vaccine. Vaccine.

[14] Massa C, Thomas C, Wang E, Marincola F, Seliger B (2015). Different maturation cocktails provide dendritic cells with different chemoattractive properties. J Transl Med.

[15] Fevzer T, Pozenel P, Zajc K, Tesic N, Svajger U. Combined TLR-3/TLR-8 signaling in the Presence of alpha-Type-1 cytokines represents a novel and potent dendritic cell Type-1, Anti-cancer Maturation Protocol. Cells 11(5) (2022). 10.3390/cells11050835 PMC8909236 35269457

[16] Garg AD, Coulie PG, Van den Eynde BJ, Agostinis P (2017). Integrating Next-Generation dendritic cell vaccines into the current Cancer Immunotherapy Landscape. Trends Immunol.

[17] Tesic N, Pekle Simonic I, Roskar K, Rozman P, Svajger U (2020). Dendritic cells generated in the Presence of platelet Lysate have a reduced type 1 polarization capacity. Immunol Invest.

[18] Kvedaraite E, Ginhoux F (2022). Human dendritic cells in cancer. Sci Immunol.

[19] ten Brinke A, van Schijndel G, Visser R, de Gruijl TD, Zwaginga JJ, van Ham SM (2010). Monophosphoryl lipid A plus IFNgamma maturation of dendritic cells induces antigen-specific CD8 + cytotoxic T cells with high cytolytic potential. Cancer Immunol Immunother.

[20] Brabants E, Heyns K, De Smet S, Devreker P, Ingels J, De Cabooter N, Debacker V, Dullaers M, JP VANM, Vandekerckhove B, Vermaelen KY (2018). An accelerated, clinical-grade protocol to generate high yields of type 1-polarizing messenger RNA-loaded dendritic cells for cancer vaccination. Cytotherapy.

[21] Vieira PL, de Jong EC, Wierenga EA, Kapsenberg ML, Kalinski P (2000). Development of Th1-inducing capacity in myeloid dendritic cells requires environmental instruction. J Immunol.

[22] Wetzel SA, McKeithan TW, Parker DC (2002). Live-cell dynamics and the role of costimulation in immunological synapse formation. J Immunol.

[23] Bakdash G, Sittig SP, van Dijk T, Figdor CG, de Vries IJ (2013). The nature of activatory and tolerogenic dendritic cell-derived signal II. Front Immunol.

[24] Samarasinghe R, Tailor P, Tamura T, Kaisho T, Akira S, Ozato K (2006). Induction of an anti-inflammatory cytokine, IL-10, in dendritic cells after toll-like receptor signaling. J Interferon Cytokine Res.

[25] Peng Q, Qiu X, Zhang Z, Zhang S, Zhang Y, Liang Y, Guo J, Peng H, Chen M, Fu YX, Tang H (2020). PD-L1 on dendritic cells attenuates T cell activation and regulates response to immune checkpoint blockade. Nat Commun.

[26] Choi Y, Sunkara V, Lee Y, Cho YK (2022). Exhausted mature dendritic cells exhibit a slower and less persistent random motility but retain chemotaxis against CCL19. Lab Chip.

[27] Bevan MJ (2004). Helping the CD8(+) T-cell response. Nat Rev Immunol.

[28] Snijders A, Kalinski P, Hilkens CM, Kapsenberg ML (1998). High-level IL-12 production by human dendritic cells requires two signals. Int Immunol.

[29] Owen KL, Brockwell NK, Parker BS, Signaling JAK-STAT. A Double-Edged Sword of Immune Regulation and Cancer Progression, Cancers 11(12) (2019) 2002. 10.3390/cancers11122002 PMC6966445 31842362

[30] Zhao T, Cai Y, Jiang Y, He X, Wei Y, Yu Y, Tian X (2023). Vaccine adjuvants: mechanisms and platforms. Signal Transduct Target Therapy.

