# Supplementary figures and images for: Factor of time in dendritic cell (DC) maturation: short-term activation of DCs significantly improves type 1 cytokine production and T cell responses
Source: J Transl Med. 2024 Jun 6;22:541. doi: 10.1186/s12967-024-05368-4 (PMC11155046; doi:10.1186/s12967-024-05368-4)

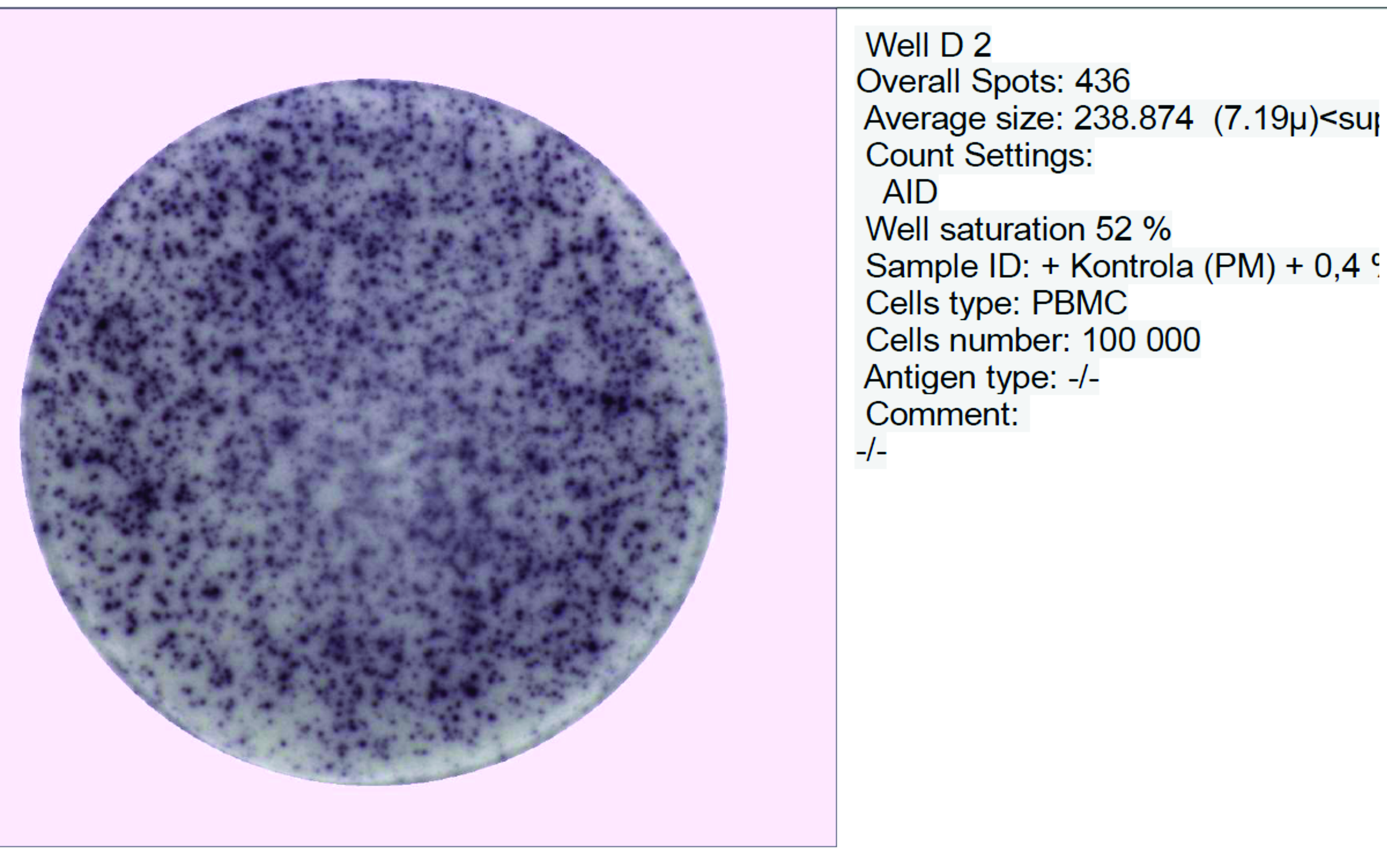

Supplement: Supplementary file 1 — Supplementary Material 1 [file 12967_2024_5368_MOESM1_ESM.jpg]
